# LOWERING THE AGE FOR MEDICARE ELIGIBILITY: WHO BENEFITS?

**DOI:** 10.1093/geroni/igad104.2089

**Published:** 2023-12-21

**Authors:** Rodlescia Sneed

**Affiliations:** Wayne State University, Detroit, Michigan, United States

## Abstract

Advocacy groups and government officials have recommended lowering the Medicare eligibility age to increase health equity and promote utilization of preventive services. Consequently, policy analysts have begun to evaluate the impact of such a policy change. The purpose of this study was to determine who would benefit most from reducing the Medicare eligibility age. Using data from the 2018 Health and Retirement Study, we calculated the number of individuals who would become insured if the Medicare eligibility age was reduced to 60, 55, or 51. We also examined health profiles for such individuals, comparing them to their insured counterparts. Complex survey procedures were used to generate population estimates. We estimate that 1.43 million Americans would become insured if the Medicare eligibility age were reduced to 60. Lowering the age to 55 or 51 would insure 3.45 million and 4.59 million people, respectively. Evaluating data for those ages 51-64, those currently uninsured reported less preventive health screening than their insured counterparts, including lower screening for high cholesterol, cervical cancer, and prostate cancer. They also had lower vaccination rates for influenza and shingles. Of those currently uninsured, 36.5% reported having no usual place where they received care. Hypertension prevalence was similar among insured and uninsured individuals, but the uninsured were less likely to be on antihypertensive medications. Taken together, our findings suggest that lowering the Medicare eligibility age would result in new healthcare coverage for individuals who underutilize the healthcare system, which could have important implications for health.

